# Socioeconomic Inequalities in Adult Obesity Prevalence in South Africa: A Decomposition Analysis

**DOI:** 10.3390/ijerph110303387

**Published:** 2014-03-21

**Authors:** Olufunke Alaba, Lumbwe Chola

**Affiliations:** 1 Health Economics Unit, School of Public Health and Family Medicine, University of Cape Town, Cape Town 7925, South Africa; E-Mail: Olufunke.Alaba@uct.ac.za; 2 PRICELESS SA, MRC/Wits Rural Public Health and Health Transitions Research Unit (Agincourt), School of Public Health, University of the Witwatersrand, Johannesburg 2050, South Africa

**Keywords:** obesity, South Africa, inequality

## Abstract

In recent years, there has been a dramatic increase in obesity in low and middle income countries. However, there is limited research in these countries showing the prevalence and determinants of obesity. In this study, we examine the socioeconomic inequalities in obesity among South African adults. We use nationally representative data from the South Africa National Income Dynamic Survey of 2008 to: (1) construct an asset index using multiple correspondence analyses (MCA) as a proxy for socioeconomic status; (2) estimate concentration indices (CI) to measure socioeconomic inequalities in obesity; and (3) perform a decomposition analysis to determine the factors that contribute to socioeconomic related inequalities. Consistent with other studies, we find that women are more obese than men. The findings show that obesity inequalities exist in South Africa. Rich men are more likely to be obese than their poorer counterparts with a concentration index of 0.27. Women on the other hand have similar obesity patterns, regardless of socioeconomic status with CI of 0.07. The results of the decomposition analysis suggest that asset index contributes positively and highly to socio-economic inequality in obesity among females; physical exercise contributes negatively to the socio-economic inequality. In the case of males, educational attainment and asset index contributed more to socio-economic inequalities in obesity. Our findings suggest that focusing on economically well-off men and all women across socioeconomic status is one way to address the obesity problem in South Africa.

## 1. Introduction

In the last two to three decades, obesity and overweight prevalence have increased globally. It is estimated that there are over one billion overweight and close to half a billion obese adults in the World [1]. Obesity has historically been a problem of developed countries, but the last few years have seen an increased epidemic in middle income countries such as South Africa (SA). Close to 30% of populations in middle income countries are either overweight or obese [1]. South Africa’s prevalence is one of the highest in sub-Saharan Africa. The 2002 South Africa Demographic and Health Survey (SADHS) reported that 29% of men and 56% of women were either overweight or obese [2]. Similar estimates are also reported in recent cross-sectional studies conducted in various settings in the country [3,4].

The increased prevalence of obesity is a major public health concern in SA, as it has short and long-term negative health consequences that may impact negatively on the country’s resources [5]. Obesity is a risk factor for health conditions such as cardiovascular diseases and some cancers [6,7,8,9], thus the increase in obesity has seen a corresponding increase in the burden of non-communicable diseases in SA and other low and middle income countries [10,11]. Managing obesity is, therefore, key to reducing the burden of non-communicable diseases and improving the health of populations. Recently, the South African National Department of Health released its 5-year strategic plan for the prevention and control of non-communicable diseases (NCDs), for 2013–2017 [12]. The strategic plan places emphasis on the need to reduce obesity and other related risk factors in order to control non-communicable diseases. However, to successfully reduce the obesity rate requires knowledge on not only the prevalence, but also its determinants and distribution in the population. There is, therefore, need to identify populations that are prone to obesity in order to establish the factors influencing the epidemic. However, information on the epidemiology of obesity in SA is scant [13]. While some studies highlight the age and gender-specific prevalence and distribution of obesity in SA [3,4], there are few studies that assess its socio-economic determinants [3,14].

Studies conducted in many high income countries show a socio-economic gradient in obesity. The prevalence of obesity is shown to be mainly concentrated in people of low socioeconomic status [15,16], probably because socioeconomic status influences energy intake and expenditure, and thus body fat [17]. Obesity is also found to be disproportionately high in economically disadvantaged women. However, the degree of income-related inequalities is declining in both men and women [18].

Hajizadeh *et al*. [19] used the concentration index (CI) to show income-related inequalities in obesity risk in Canada. The study found that obesity was concentrated in rich men and economically disadvantaged women, and that the degree of socioeconomic related inequalities was reducing over time. Ljungvall and Gerdtham [20] studied the trend in income-related inequalities in obesity in Sweden, and showed that obesity inequalities were concentrated among the rich. The authors recommended that reducing income inequalities would be the most effective strategy to reduce obesity inequalities. Costa-Font and Gil [21] also found evidence of socioeconomic inequalities in obesity in Spain. Using a decomposition analysis, they showed that education and demographic factors were some of the main contributors to inequalities in obesity. Nikolaou and Nikolaou [18] examined the income-related inequalities in obesity in 10 European countries, using the European Community Household Panel dataset. The study found that income-related inequalities in Europe were more concentrated in economically disadvantaged women.

In low and middle income countries, obesity has been shown to be mainly prevalent among populations of high socioeconomic position, which are increasingly adopting Western lifestyles and diets [22]. It can be shown that as economies transition from low to middle and high income, obesity trends shift from being concentrated among society’s well off to the poor [23,24]. We would expect the obesity trends in SA and other middle income countries to follow the Western pattern, but this is not certain. It is, therefore, important to monitor the trends in obesity and the related socioeconomic gradient. A thorough understanding of trends in the relationship between socioeconomic status and overweight and obesity prevalence will provide useful insights for developing effective overweight intervention programmes and policies. We did not find any published literature on the formal measurement of the socioeconomic gradient of obesity in SA. This study thus aims to fill this gap.

In this study, we examine the socioeconomic inequalities in obesity prevalence in South African adults. We use the CI to examine the relationship between socioeconomic status and obesity. The CI, an approach for studying inequality used in economics, has proved useful to understanding the socioeconomic gradient of obesity, as well as other health factors [15,20,25]. The CI is a measure of association which indicates the degree to which a factor, such as obesity, varies with a measure of household resources. The CI is most useful because it not only provides a single index of wealth related inequality in obesity, but can also be used in decomposition analysis to examine the underlying causes of the wealth related inequality. Thus in this study, we also perform a decomposition analysis, in order to determine the factors driving socioeconomic inequalities in obesity.

## 2. Methods

### 2.1. Data

Our investigation is based on the adult questionnaire of the first wave of the South African National Income Dynamics Study (SA-NIDS) version 5.0. The SA-NIDS is a nationally representative survey undertaken in 2008 by the South African Labour and Development Research Unit (SALDRU) based at the University of Cape Town (UCT). The survey is a panel study that documents the dynamic structure of household members and changes in their incomes, expenditures, assets, access to services, education, health and other dimensions of well-being. A household questionnaire, together with an adult questionnaire, was administered to every household member aged 15 years and older. The survey consisted of 16,800 adults. In our study, the unit of analysis is an individual, classified as an adult above 18 years. After cleaning the data and excluding individuals less than 18 years, pregnant women and those with missing body mass index (BMI), our sample reduced to 11,326 individuals; the analysis was further disaggregated by sex with 4,983 males and 6,343 females. A detailed report on the SA-NIDS methodology is provided elsewhere [26].

### 2.2. Measures

#### 2.2.1. Health Indicators

Obesity—the heights and body weight of adults were taken during the SA-NIDS survey. These were used in our analysis to compute the body mass index (BMI). Body mass is defined as a person’s weight in kilogrammes divided by the square of height in meters. Obesity is defined as BMI greater or equal to 30 kg/m^2^ [27]. In our analysis, obesity was measured as a binary variable with (1) obese and (0) not obese.

#### 2.2.2. Measurement of Inequality

There are various measures of inequality, but as suggested by Wagstaff *et al*. [28], any inequality measure is expected to meet three minimal requirements: (1) the measure should reflect the experiences of the whole distribution of the population rather than the extremes of the social class; (2) the measure should be sensitive to changes in the distribution of the population across socioeconomic groups; and (3) the measure should exhibit socioeconomic features to inequalities in health.

The CI is among the few measures that satisfy this criteria [28]. In light of its usefulness not only to quantifying health inequalities, but also to decomposing the contribution of various factors to health inequalities, we used the CI to examine socioeconomic related inequality in the distribution of obesity across the adult population of SA.

The CI is used to examine relative inequality in health and its values lie between negative one (−1) and positive one (+1). Negative values imply that the health measure used (obesity in this paper) is more concentrated among the poorer socioeconomic population. Positive values imply that obesity is more concentrated among the richer population. Larger absolute values of CI indicate wider inequalities in obesity. A CI value of zero signifies that obesity is equally distributed across socioeconomic status [29,30].

The CI can be defined simply as twice the covariance between the health variable (*y*: obesity) of individual *i* and the ranking of the socioeconomic status, *r*, divided by the mean of the health variable (*μ*):

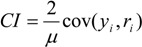
(1)

As suggested by Kakwani *et al.* [31], the CI can also be computed easily by what is called the “convenient regression approach”. This method of calculation has the advantage of not only yielding an estimate of CI, but also generating standard errors from which statistical inferences can be produced. This can be written as follows:

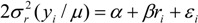
(2)
where 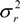 is the variance of the rank (r) , while the other variables remains as defined in Equation (1), *β* is an estimated concentration index, and *ε* is the stochastic error term. This CI yields what is called an unstandardized concentration index.

Standardization is an adjustment technique that is used to account for differences in population demographic structures, to produce a refined description of the relationship between health and socioeconomic status [30], and also to facilitate comparisons for different populations, sub-populations or overtime. Standardization is best utilized under the assumption that demographic variables such as sex and age are correlated with either the health measure, socioeconomic status, or both [30]. For instance, older people tend to have higher levels of body weight [32,33] and women have much more peripheral body fats in the legs and hips than men [21]. There are two ways of standardizing: the direct and indirect methods [30]. The indirect standardization method is preferred and commonly used over direct standardization because of its greater accuracy when dealing with individual-level data [30]. The indirect standardization method is used in this paper.

Although the estimate of CI can be indirectly standardized by subtracting the influence of all standardizing variables from the unstandardized CI [30], an equivalent approach of obtaining an indirectly standardized CI is to include the standardizing variables directly into the “convenient regression”, either for full or partial correlations of the health variable, with the standardizing variables. In the case of the former, only the standardizing variables, which can also be seen as confounding variables are included in the regression while in the latter case, other non-confounding variables are included in order to estimate the correlation of the confounding variables conditional on those other variables [30]. This procedure was used to standardize obesity in this paper.

The indirect standardized health variable (in this case obesity) *ŷ_i_^is^* is attained by a simple regression on actual health variable (*y*) of individual *i* as follows:

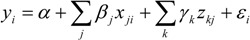
(3)
where *x_j_* are the confounding variables for which we want to age-standardize individual *i* (age is the only standardizing variable used in our study, due to the fact that there was a split in the analysis: females and males); *α*, *β* and *γ* are the parameter vectors, the *z_k_* are the non-confounding variables for which we do not want to standardize, but to control for, in order to estimate partial correlations with the confounding variables, and *ε* is the error term. To standardize for full correlations, non-confounding variables, *z_k_* are excluded from the regression. The *z_k_* variables in our study include education, employment status, race, asset index, marital status, and behavioural indicators: exercise and smoking.

The Ordinary Least Squares (OLS) parameter estimates from Equation (3) are then used to obtain the predicted values of the health indicators as expressed below:

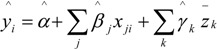
(4)

The estimate of the indirectly standardized obesity (*ŷ_i_^is^*) is the difference between actual health measure (*y_i_*) and x-expected health (*ŷ_i_*), plus the sample mean (*y*) as expressed in Equation (4):

*ŷ_i_^is^* = *y_i_* − *ŷ_i_^x^* + *y* (5)

We can therefore infer that the distribution of *ŷ_i_^is^* across socioeconomic groups is the distribution of obesity in the female and male populations that would be expected, irrespective of differences in the distribution of age.

Given that our health measure was binary (1,0), the bounds of the concentration index are not −1 and 1 but depend on the mean of the variable [34,35]. A normalization process that ensures that the CI is quantified in the range −1 to 1, for any given mean of the health measure (obesity) is therefore recommended, by multiplying the calculated concentration index by (1/1 − μ) [34]. Recently, there has been a debate regarding the appropriate normalization process between Wagstaff [34,35] and Erreygers (ehe Erreygers index (E_c_) can be stated as (4μ/b − a)·CI, where, *a* and *b* are upper and lower limits of the health variable, CI is the standard concentration index, and *μ* is the mean of the health variable) [36,37]. Kjellson and Gerdtham [38] give a detailed discussion on the differences between inequality indices and state that the normalization choice should be based on the researcher’s value judgment. Also of note is that Erreygers index does not change the direction of inequality, but only the magnitude. Erreygers index can also be obtained by scaling what is obtained from Wagstaff’s normalization by the factor (4μ/1 − μ) [39]. We therefore, decided to apply the normalization process proposed by Wagstaff (we obtained similar results using both Wagstaff and Erreygers normalization index).

#### 2.2.3. Explaining Inequality: Decomposition of the Concentration Index

The CI can be decomposed into the contributions of explanatory factors using regression analysis, thereby allowing for an analysis of the contribution of each individual factor to the measured degree of socio-economic inequalities in health [40]. However, the method relies on the linearity of the underlying regression model, and in cases where the health outcome is binary in nature, whose ideal specification is nonlinear, a decision on whether to approximate the model by a linear specification using linear probability model (LPM) or to approximate the decomposition of the CI [41] is needed. We chose to approximate the model by applying a linear probability model on the health outcome because the approximation of the decomposition of the CI generates a non-unique result [30], which captures the linear association between the health variable and the covariates. This linearity in parameters is a useful property for the decomposition of the inequality index [30,40,41]. Moreover, previous studies that measure inequality and decomposition of inequality show that while estimations produced by linear probability models may be less robust and precise than those generated by non-linear models, they both yield similar results and the estimated parameters of LPM are generally consistent [30,42].

Therefore, given a linear additive regression model of individual health y expressed as: 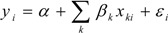, the CI can be written as follows to include the elasticities and inequalities of the various determinants [30,40]:

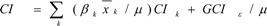
(6)
where *μ* is the mean of the health variable (*y*: obesity in this case), the index *k* refers to the regressors included in the obesity equation; *β_k_* is the coefficient for each of the health determinants from equation 6, *x_k_* is the mean of each of the regressors, *GCI_s_* is the generalized concentration index for the error term (*ε*) [30].

The component *β_k_*·*x_k_*/*μ*, is defined as elasticity (*η_k_*) of with respect to *x_k_* [30] and measures the impact of each covariate on obesity and *CI_k_* is the concentration index for each of the individual regressors; it measures the degree of unequal distribution across socioeconomic status. We applied the Wagstaff normalization not only to the concentration index but also to the decomposition.

#### 2.2.4. Construction of the Living Standard Measure: Asset Index

Various ways of measuring living standards are used in literature—income, expenditure and wealth index. In developing countries, the use of the measure of income and consumption are prone to various bias including recall bias [43], variation of income from season to season and reluctance to divulge information [44]. It should be noted that socioeconomic inequalities in a health variable may be sensitive to the choice of welfare indicator, depending on the health variable being examined. Wagstaff and Watanbe [45] found that the concentration indices for health care utilization were more sensitive to the choice of welfare indicator but not to the health outcome (child malnutrition). Also of note is the fact that the CI reflects the relationship between the health variable and living standards rank and not the variance of the living standards measures [30].

In this paper, the household assets index [46,47] is the main socioeconomic indicator used (there are other indicators of socioeconomic status like education, employment status, composite socioeconomic status among others, indicating positions in the society or as defined by Oakes and Rossi [48]: “…differential access (realized and potential) to desired resources”). However, the measurement still lacks conceptual clarity, and the use depends on the question being asked by the researcher. In this paper, given that we are analysing a concentration index, a continuous variable is needed as a measure of wellbeing). The asset index was constructed using multiple correspondence analysis (MCA) [49], a preferred technique for categorical variables [50] expressed as follows:

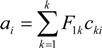
(7)
where *α_i_* is the value of the asset index for the *i*th observation, *c_ki_* is the value of the *k*th dummy/categorical variable (for *k* = 1,….K) describing the set of six household assets and living conditions considered in the analysis, which included type of dwelling, access to water, access to toilet, radio, cooking gas and possession of a cellular phone. None of these variables contained negative values. *F*_1*k*_ is the asset index weight of the first component of the analysis. The resulting asset scores generated were used to rank the population from lowest to the highest, and to rank and divide the population into wealth quintiles (from 1-lowest to 5-highest).

Although, there are various methods of constructing asset indices such as principal components analysis (PCA) [46] and factor analysis [51], MCA was chosen for this analysis because it is better suited when there is a combination of binary and categorical variables, it avoids previous computations of “squeezing” categorical assets into binary as needed in the framework of PCA [50,52].

#### 2.2.5. Other Variables

Apart from socioeconomic status measured by asset index, a range of variables, which studies have shown to influence BMI and also the socioeconomic gradient in obesity rates, were included in estimating the CI and the regression model used for the decomposition analysis [15,20,21]. These include: Education, measured in years of schooling and categorized as 1-no education, 2-primary with 1–7 years, 3-secondary with 8–12 years and 4-tertiary, with 13+ years of schooling; Employment status, a binary variable with 1-employed and 0-unemployed, the unemployed consists of all those who are not in formal or informal employment; Marital status, was coded 1-never married 2-married/cohabiting and 3-divorced/widowed/separated; and Area of residence, a binary variable with 1-rural and 0-urban. Race was included as a categorical variable with 1- African, 2-coloured, 3-Asian and 4-Whites. The South African population is made up of 79.8% African, 8.9% Coloured, 2.6% Indian/Asian and 8.7% White people, according to the 2013 mid-year population estimates [53].

Lifestyle factors, including diet, physical activity, the use of tobacco and alcohol, have been identified as risk factors for obesity [19]. We included two lifestyle factors namely physical exercise, a dummy variable (1 if an individual exercises at least once a week) and smoking (1 if an individual smokes, 0 otherwise). Since obesity is essentially an imbalance between calorie intake and expenditure, the inclusion of information on physical activity is important. Although there is high probability that the inclusion of the lifestyle factors could be endogenous, we strongly believe in the importance of these factors in the decomposition analysis not only because of the demonstrated association of these factors with obesity and obesity inequality in the literature [54] but also because they can be classified as “policy- relevant variables” in the context of the measurement of socioeconomic related inequality in health or healthcare (Gravelle estimated a directly standardized partial concentration index, and suggests the use of three types of variables in the regression equation used for the decomposition analysis, namely: income, need standardizing variables and other possible policy-relevant variables [55]). Consequently, our estimated regression model can be viewed as a reduced form demand model for obesity and no causal interpretation is implied.

### 2.3. Data Analysis

Management of data and analysis were done in Stata 12 (Stata Corp. Inc., College Station, Texas, USA) and ADePT version 5.0, which was developed by the World Bank and specifically designed for analyzing inequality in health outcomes and related research [56]. Chi-squared (χ^2^) significance tests were used to assess differences between quintiles in the distribution of obesity for men and women, and OLS regression was applied in the decomposition analysis. The SA-NIDS was a multistage sampling procedure, thus all estimates took into account the sampling weights and adjustment was made for clustering and stratification of the survey data.

## 3. Results

### 3.1. Descriptive Statistics

Table 1 shows the socio-demographic characteristics of the study sample by gender. There were 11,326 adults included in the sample, of which 56% were female. The age-standardized obesity was 35% among females and 12% among males, and approximately 25% of the total population was obese.

**Table 1:** Socio-demographic characteristics, health and lifestyle measures.

Variables	Male 4,983 (44%)	Female 6,343 (56%)	Total 11,326
Unstandardized obesity (age-standardized * )	11% (12%)	36% (35%)	24% (25%)
Age in years (standard deviation)	37 (13.9)	39 (16.1)	38 (15.2)
***Marital status***			
Married	46%	44%	45%
Widowed	5%	15%	10%
Never married	49%	41%	45%
***Education***			
No school	7%	11%	9%
Primary	21%	21%	21%
Secondary	60%	58%	59%
Tertiary	12%	10%	11%
***Employment status***			
Employed	60%	38%	43%
Unemployed	40%	62%	52%
***Residence***			
Urban	64%	59%	61%
Rural	36%	41%	39%
***Race***			
African	81%	80%	80%
Coloured	7%	8%	8%
Asian	2%	2%	2%
White	10%	10%	10%
***Lifestyle factors***			
Physical exercise	40%	20%	28%
Smoking	40%	9%	23%

Note: * This is the indirectly standardized obesity, for age-only.

The mean age was around 38 years (SD = 15.2). The majority of respondents had secondary (59%), followed by primary level (21%) and only a few had tertiary (11%) education. The proportion of unemployed persons was 52%. About 36% of the females and 60% of the males were employed. The proportion of married (45%) and never married (45%) were equally distributed in the population, and about 11% were widowed. The majority of the population lived in urban areas (61%).

### 3.2. Inequalities in Obesity

#### 3.2.1. Wealth Distribution

**Figure 1:**
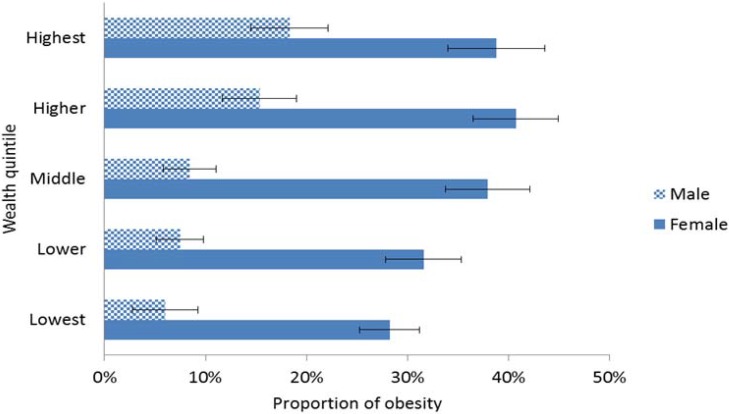
Distribution of unstandardized obesity by wealth and gender.

Note: The error bars in Figure 1 represent 95% confidence intervals.

Figure 1 shows the distribution of unstandardized obesity by wealth (as measured by the asset index) and gender. The proportion of obesity was about 20% in the lowest quintile, compared to 30% in each of the highest two. Generally, there was a positive relationship between obesity and wealth for both men and women (*p* < 0.05). Obesity was more pronounced in females, ranging from 28% in quintile 1 to 39% in quintile 5 and 41% in quintile 4. Among males, obesity steadily increased with socioeconomic status from 6% in the lowest quintile to 18% in the highest quintile.

#### 3.2.2. Concentration Indices

The normalized concentration indices for males and females are given in Table 2. Included in the table are three indices: (1) the unstandardized, (2) indirectly standardized for age only and (3) indirect standardized for age and other non-confounders. The 3 methods produced different but similar figures, however, to be consistent with our decomposition analysis, all discussions herewith are based on the third index—age including non-confounding variables. The indices were all positive, indicating that obesity was more concentrated among the rich. The normalized concentration index was 0.12 for the entire population, 0.09 for females and 0.27 for males. This indicates that there is more inequality in the distribution of obesity among men compared to women. Though obesity is mainly found among the wealthy, the distribution of obesity is not very different between poor and rich women. However, richer men are more likely to be obese than their poorer counterparts.

**Table 2:** Concentration indices of adult obesity.

Method	Female		Male		Total	
Standardization method	CI	95% confidence interval	CI	95% confidence interval	CI	95% confidence interval
Unstandardized	0.11	(0.06–0.16)	0.28	(0.18–0.38)	0.13	(0.009–0.17)
Age standardized only	0.13	(0.05–0.19)	0.26	(0.17–0.35)	0.13	(0.09–0.17)
Age and non–confounding variables	0.09	(0.03–0.14)	0.27	(0.17–0.36)	0.12	(0.06–0.17)

Notes: All CIs are Wagstaff normalized indices. CI = Concentration Index. Non-confounding variables include: Socioeconomic status (asset index), marital status, education, employment, residence, race and lifestyle factors.

### 3.3. Explaining Obesity Inequalities

#### 3.3.1. Results of the Linear Regression

Table 3 shows the results of the linear probability regression model. Age and socioeconomic status (measured by the asset index) were statistically significant and positively associated with obesity among females, while the positive factor associated with obesity in males was higher education. Marital status was significantly associated with obesity. For both genders, the single and never married were less likely to be obese than married people. Increased physical activity was associated with low obesity in women. Smoking was a significant negative factor for obesity for men as well as women.

**Table 3:** Results of the linear probability regression model.

Variables		Female		Male	
		Coefficients	Standard Error	Coefficients	Standard Error
Age		**0.008**	0.00	**0.003**	0.00
Socioeconomic status (asset index)		**0.051**	0.01	0.018	0.01
***Employment Status***					
Unemployed		(base)			
Employed		**0.050**	0.02	**0.034**	0.01
***Education***					
No school		(base)		(base)	
Primary		**0.094**	0.03	0.058	0.02
Secondary		**0.098**	0.03	**0.096**	0.03
Tertiary		0.058	0.05	**0.132**	0.04
***Marital status***					
Married		(base)		(base)	
Widowed		−0.048	0.03	−0.073	0.04
Never married		**−0.079**	0.02	**−0.049**	0.02
***Area of residence***					
Urban		(base)		(base)	
Rural		−0.028	0.02	−0.005	0.01
African		(base)			
Coloured		−0.021	0.04	0.015	0.03
Asia/India		−0.124	0.07	0.050	0.09
White		−0.036	0.05	**0.070**	0.04
Physical activity		**−0.098**	0.03	**−0.036**	0.02
Smoking		**−0.107**	0.04	**−0.058**	0.01
Intercept		0.024	0.05	−0.062	0.05
Observations		6816		4510	
R^2^			0.88		0.09

Note: Coefficients significantly different from zero (at *p* < 0.05) are in bold typeface.

#### 3.3.2. Decomposition of Socioeconomic Inequality in Obesity

Table 4 presents the decomposition analysis based on OLS regressions, indicating the elasticity, concentration index (Wagstaff normalized) and contribution of each covariate to the overall obesity inequality. If the contribution of a factor is positive, this can be interpreted as *ceteris paribus*, the obesity inequality would be lower if that factor was not present (the opposite for negative contributions).

**Table 4:** Decomposition of concentration indices for women and men.

Variables	Female						Male					
	Elasticity		CI		Contribution		Elasticity		CI		Contribution	
Age	0.865		0.003		0.000 (0%)		1.080		0.021		0.023 (8.5%)	
Socioeconomic status (asset index)	0.028		2.614		0.054 (71.4%)		0.045		1.767		0.067 (24.8%)	
***Employment Status***												
Unemployed	(base)		(base)		(base)		(base)		(base)		(base)	
Employed	0.054		0.124		0.007 (10.0%)		0.184		0.100		0.018 (6.6%)	
***Education***												
No school	(base)		(base)		(base)		(base)		(base)		(base)	
Primary	0.055		−0.179		−0.010 (−14.3%)		0.107		−0.247		−0.026 (−9.6%)	
Secondary	0.159		0.082		0.013 (18.6%)		0.527		0.060		0.032 (11.9%)	
Tertiary	0.017		0.450		0.008 (11.4%)		0.141		0.466		0.066 (24.4%)	
***Marital status***												
Married	(base)		(base)		(base)		(base)		(base)		(base)	
Widowed	−0.021		−0.026		0.001 (1.4%)		−0.030		−0.004		0.000 (0.0%)	
Never married	−0.089		−0.059		0.005 (7.1%)		−0.219		−0.066		0.014 (5.2%)	
***Area of residence***												
Urban	(base)		(base)		(base)		(base)		(base)		(base)	
Rural	−0.031		−0.453		0.014 (20.0%)		−0.017		−0.447		0.008 (3.0%)	
Black	(base)	(base)		(base)		(base)		(base)		(base)	
Coloured	−0.005	0.43		−0.002 (−2.9%)		0.010		0.359		0.003 (1.1%)	
Asian/Indian	−0.008	0.66		−0.005 (−7.1%)		0.010		0.501		0.005 (1.9%)	
White	−0.10	0.67		−0.007 (−10.0%)		0.061		0.645		0.040 (14.8%)	
***Lifestyle***											
Physical activity	−0.054	0.370		−0.020 (−28.6%)		−0.130		0.102		−0.013 (−4.8%)	
Smoking	−0.028	0.290		−0.008 (−11.4%)		−0.209		−0.023		0.005 (1.9%)	
Residual *				0.017						0.029	

Notes: CIs are Wagstaff normalized indices. CI = Concentration Index. We also performed the analysis by excluding lifestyle variables as a check, and the size and magnitude of all variables except socioeconomic status (which increased) did not change. * Residual is the part of socioeconomic related inequality not explained by the chosen determinants.

Low education is concentrated among the poor in both males and females. The widowed and never married were concentrated in low-income groups among females, but only singles were in this group among men. Lifestyle factors consisting of physical exercise and smoking had positive indices for women, implying that these activities were concentrated among wealthy individuals. Among men, smoking was more an attribute of the poor.

The highest elasticities for both men and women were observed with age. However, age did not make a large contribution to the overall concentration index. The largest contributor to the obesity inequality for both men and women was socioeconomic status (asset index). Socioeconomic status contributed to about 71.4% of the inequalities among women, compared to 24% among men. Education was a major contributor to the obesity inequality among men, but not women. Tertiary education contributed to about 24.4% of the inequality for men. Other notable drivers of inequalities among men were age (8.5%) and employment (6.6%). Living in a rural area (20.3%), physical activity (−28.6%) and smoking (−11.4%) contributed significantly to the obesity inequality among women, but not men.

## 4. Discussion

This paper provides evidence of the socioeconomic inequalities in obesity among South African adults. To our knowledge, this is the first study to formally measure the socioeconomic gradient of obesity in SA using the concentration index (CI). We use data from the 2008 South African National Income Dynamics Study (SA-NIDS) to calculate CIs for men and women, and apply a decomposition analysis to estimate the contributors to obesity inequality. An advantage of CI is that it is more sensitive to changes in the socioeconomic distribution. A major limitation of CI is that it may only be applied if a strict ranking socioeconomic variable, such as income or consumption is available. However, it has been shown that the asset index provides a reasonable proxy for measuring inequalities in the absence of income or consumption [57]. Including the behavioral factors in the decomposition may introduce bias in the study due to endorgeneity. For instance, physical exercise is endogenous because causality cannot be identified with regard to whether people who exercise have a lower probability of being obese or people who are not obese are more likely to exercise [58]. In our analysis, we tested the effect of the lifestyle factors and did not find any significant differences when they were removed from the regression analysis. Thus we included them on the basis that they are important to informing policy decisions on obesity prevention [55], and also because they have been shown to be associated with obesity in the literature [59].

The use of a large nationally representative sample made it possible to examine relationships between obesity and its socioeconomic determinants, and measure obesity inequalities. The height and weight scores used to estimate obesity in the SA-NIDS were measured and not self-reported, hence we are confident that errors in measurement were minimal. Causal inference on the determinants of obesity is, however, limited by the cross-sectional nature of the study. Therefore, a comprehensive longitudnal study is required to provide a more detailed description of the reasons for changes in weight [20].

The rapid economic growth experienced by many low and middle income countries in the last few years has seen a parallel nutrition transition, with diets increasingly constituting greater amounts of sugar, fats and refined carbohydrates [60]. This has exarcerbated excess weight gains for both men and women. Our study, however, suggests some gender disparities in the distribution of obesity, showing that obesity is higher in women than men. This is consistent with findings from other studies in SA [3,4], and other developing countries [61], which show that women tend to be more obese than men.

The study shows that unlike in many developed countries where the socioeconomic gradient in obesity is concentrated among the poor [15,16], obesity in SA is positively related to wealth, with well-off individuals more likely to be obese than the poor. This is consistent with findings from other studies in low and middle income countries that show that obesity tends to increase with higher socioeconomic status [22].

While socioeconomic status (measured by the asset index) was a major contributor to obesity in both men and women, the concentration index of obesity in women was small, indicating that there were less inequalities in the distribution of obesity for women across socioeconomic status. This was different for men, where wealthier men were significantly more likely to be obese than their poorer counterparts. The decomposition analysis demostrates that other measures of socioeconomic position, including employment and higher education were major contributors of inequalities in obesity for men, but not for women. Lifestyle factors of physical activity and smoking, and living in a rural area had a higher impact on obesity inequalities in women than men.

There could be various reasons for these disparities in the distribution of obesity inequalities among women and men, which are not analysed here, but should be analysed in future studies. Firstly, though the nutrition transition observed in low and middle income countries has contributed to excess weight gain in both genders, it has had a higher impact on women and wealthier men, particularly on their physical activities [62]. There could be high physical inactivity related to both employment and unemployment or underemployment. Increased economic activity in SA has led to higher employment among men, particularly in professional, managerial, or administrative work that promote sedentary behaviour. Wardle *et al.* [63] show that men with low socioeconomic status are more likely to be employed in work that is physically demanding, thus may have a lower risk of obesity than men who are well off. Women on the other hand, are more likely to be caregivers or in wage labour that does not require vigorous physical activity [64]. Further, women as caregivers are mainly responsible for preparation of food and meals in the home. With the proliferation of kiosks, supermarkets and fastfood restaurants, there is an increased accessibility of food [65] which does not require expended energy and effort to prepare, contributing to reduced physical activity.

In this study, we find that rural women are less likely to be obese than women living in urban areas, and area of residence was a significant contributor to obesity inequalities, particularly among women. This could be due to the impact of the nutrition transition, which tends to be higher in urban areas, as well as the fact that the role that rural women play in the home and also their employment, mainly in argrarian work, requires vigorous physical activity [66].

Cultural issues may also play a significant role in obesity, and this might be a reason why the socioeconomic gradient in obesity among women was small. Particularly among the black South African population, who are in the majority, larger body size is considered to be a sign of beauty, prosperity and good health [67]. These views on body image may be a major contributor to overweight and obesity among women of all socioeconomic backgrounds, and wealthy men.

## 5. Conclusions

This paper provided an analysis of the socioeconomic gradient in obesity in the South African adult population. The study shows that obesity is significantly higher in women than men. The results also show that the socioeconomic gradient in obesity favours the rich. However, the gradient is more pronounced in men than women. A regression-based decomposition of wealth related inequality in obesity was also performed, showing that while the main contributor is socioeconomic status (measured by asset index) for both men and women; other main contributors to obesity inequalities are economic factors of education and employment among men, and lifestyle factors of physical activity among women.

These findings have wider implications for policy and future research on obesity. There is need for more research on the socioeconomic gradient in adult obesity, which will help understand the cause of the obesity epidemic and facilitate the reduction in health disparities. The fact that the prevalence of obesity in women is high should be of concern, because obese women are likely to give birth and raise children who might become obese or overweight [68]. Thus focusing on reducing obesity in women could benefit future generations.

Promotion of physical activity, particularly among women is very important to the reduction of obesity inequality. Based on our findings, we suggest that policies aimed at preventing obesity in women should be population based, and target women across the socioeconomic spectrum. There is also need for workplace activities that promote physical activity, better nutrition and generally improved lifestyle and living. These issues are acknowledged as important to reducing NCDs and their risk factors, such as obesity, in the SA Department of Health NCDs strategic plan [12].

## References

[1] WHO (2010). Global Status Report on Noncommunicable Diseases.

[2] Puoane T., Steyn K., Bradshaw D., Laubscher R., Fourie J., Lambert V., Mbananga N. (2002). Obesity in South Africa: The South African demographic and health survey. Obes. Res..

[3] Malhotra R., Hoyo C., Østbye T., Hughes G., Schwartz D., Tsolekile L., Zulu J., Puoane T. (2008). Determinants of obesity in an urban township of South Africa. S. Afr. J. Clin. Nutr..

[4] Malaza A., Mossong J., Barnighausen T., Newell M.L. (2012). Hypertension and obesity in adults living in a high HIV prevalence rural area in South Africa. PLoS One.

[5] Mayosi B.M., Flisher A.J., Lalloo U.G., Sitas F., Tollman S.M., Bradshaw D. (2009). The burden of non-communicable diseases in South Africa. Lancet.

[6] Kotchen T.A. (2010). Obesity-related hypertension: Epidemiology, pathophysiology, and clinical management. Amer. J. Hypertens..

[7] Marinou K., Tousoulis D., Antonopoulos A.S., Stefanadi E., Stefanadis C. (2010). Obesity and cardiovascular disease: From pathophysiology to risk stratification. Int. J. Cardiol..

[8] Nguyen N., Nguyen X.-M., Lane J., Wang P. (2011). Relationship between obesity and diabetes in a US adult population: Findings from the national health and nutrition examination survey, 1999–2006. Obes. Surg..

[9] Wolin K.Y., Carson K., Colditz G.A. (2010). Obesity and cancer. Oncologist.

[10] Aikins A.G., Unwin N., Agyemang C., Allotey P., Campbell C., Arhinful D. (2010). Tackling Africa’s chronic disease burden: From the local to the global. Globalization Health.

[11] Parkin D.M., Sitas F., Chirenje M., Stein L., Abratt R., Wabinga H. (2008). Part I: Cancer in indigenous Africans—Burden, distribution, and trends. Lancet Oncol..

[12] (2013). National Department of Health: Strategic Plan for the Prevention and Control of Non-communicable Diseases 2013–2017.

[13] Goedecke J.H., Jennings C.L., Lambert E.V., Steyn K., Fourie J., Temple N. *Obesity in South Africa*. Chronic Diseases of Lifestyle in South Africa: 1995–2005.

[14] Senekal M., Steyn N.P., Nel J.H. (2003). Factors associated with overweight/obesity in economically active South African populations. Ethn. Dis..

[15] Zhang Q., Wang Y. (2007). Using concentration index to study changes in socio-economic inequality of overweight among US adolescents between 1971 and 2002. Int. J. Epidemiol..

[16] Markwick A., Vaughan L., Ansari Z. (2013). Opposing socioeconomic gradients in overweight and obese adults. Aust. N. Z. Publ. Health.

[17] Brennan S.L., Henry M.J., Nicholson G.C., Kotowicz M.A., Pasco J.A. (2009). Socioeconomic status and risk factors for obesity and metabolic disorders in a population-based sample of adult females. Prev. Med..

[18] Nikolaou A., Nikolaou D. (2008). Income-related inequality in the distribution of obesity among Europeans. J. Public Health.

[19] Hajizadeh M., Campbell M.K., Sarma S. (2014). Socioeconomic inequalities in adult obesity risk in Canada: Trends and decomposition analyses. Euro. J. Health Econ..

[20] Ljungvall A., Gerdtham U.G. (2010). More equal but heavier: A longitudinal analysis of income-related obesity inequalities in an adult Swedish cohort. Soc. Sci. Med..

[21] Costa-Font J., Gil J. (2008). What lies behind socio-economic inequalities in obesity in Spain? A decomposition approach. Food Policy.

[22] Steyn K., Damasceno A., Jamison D.T., Feachem R.G., Makgoba M.W., Bos E.R., Baingana F.K., Hofman K.J., Rogo K.O (2006). Lifestyle and Related Risk Factors for Chronic Diseases. Disease and Mortality in Sub-Saharan Africa.

[23] Monteiro C.A., Moura E.C., Conde W.L., Popkin B.M. (2004). Socioeconomic status and obesity in adult populations of developing countries: A review. Bull. WHO.

[24] Sobal J., Stunkard A. (1989). Socioeconomic status and obesity: A review of the literature. Psychol. Bull..

[25] Nkonki L.L., Chopra M., Doherty T.M., Jackson D., Robberstad B. (2011). Explaining household socio-economic related child health inequalities using multiple methods in three diverse settings in South Africa. Int. J. Equity Health.

[26] Leibbrandt M., Woolard I., de Villiers L. (2009). Methodology: Report on NIDS Wave 1.

[27] (1995). Physical Status: The Use and Interpretation of Anthropometry. Technical Report Series 854 1-1-9950.

[28] Wagstaff A., Paci P., Doorslaer E.V. (1991). On the measurement of inequalities in health. Soc. Sci. Med..

[29] Wagstaff A., Doorslaer E.V., Doorslaer E.V. (1993). Equity in the Finance and Delivery of Health Care: Concepts and Definitions. Equity in the Finance and Delivery of Health Care an International Perspective.

[30] O’Donnell O., Doorslaer E.V., Wagstaff A., Lindelow M. (2008). Analyzing Health Equity Using Household Survey Data: A Guide To Techniques And Their Implementation.

[31] Kakwani N., Wagstaff A., Doorslaer V. (1997). Socioeconomic inequalities in health : Measurements, computation, and statistical inference. J. Econ..

[32] Baum C., Ruhm C.J. (2009). Age, socioeconomic status and obesity growth. J. Health Econ..

[33] Costa-Font J., Joan G. (2004). Social interactions and the contemporaneous determinants of individuals’ weight. Appl. Econ..

[34] Wagstaff A. (2005). The bounds of the concentration index when the variable of interest is binary, with an application to immunization inequality. Health Econ..

[35] Wagstaff A. (2009). Correcting the concentration index: A comment. J. Health Econ..

[36] Erreygers G. (2009). Correcting the concentration index. J. Health Econ..

[37] Erreygers G., van Ourti T. (2011). Putting the cart before the horse. A comment on Wagstaff on inequality measurement in the presence of binary variables. Health Econ..

[38] Kjellsson G., Gerdtham U.-G. (2013). On correcting the concentration index for binary variables. J. Health Econ..

[39] Ataguba J.E., Akazili J., McIntyre D. (2011). Socioeconomic-related health inequality in South Africa: Evidence from general household surveys. Int. J. Equity Health.

[40] Wagstaff A., van Doorslaer E., Watanabe N. (2003). On decomposing the causes of health sector inequalities with an application to malnuitrition inequalities in Vietnam. J. Econometrics.

[41] Van Doorslaer E., Koolman X., Jones A. (2004). Explaining income-related inequalities in doctor utilization in Europe. Health Econ..

[42] Van Doorslaer E., Masseria C., OECD Health Equity Research Group (2004). Income-related Inequality in the Use of Medical Care in 21 OECD Countries. Towards High-performing Health Systems: Policy Studies Paris.

[43] Sahn D., Stifel D. (2003). Exploring alternate measures of welfare in the absence of expenditure data. Rev. Income Wealth.

[44] Howe L.D., Hargreaves J.R., Huttly S.R. (2008). Issues in the construction of wealth indices for the measurement of socio-economic position in low-income countries. Emerg. Themes Epidemiol..

[45] Wagstaff A., Watanabe N. (2003). What difference does the choice of SES make in health inequality measurement?. Health Econ..

[46] Filmer D., Pritchett L.H. (2001). Estimating wealth effects without expenditure data-or tears: An application to educational enrollments in states of India. Demography.

[47] Boysen F., van der Berg S., von Maltitz M., du Rand G. (2008). Using an asset index to assess trends in poverty in seven sub-Saharan African countries. World Develop..

[48] Oakes J.M., Rossi P.H. (2003). The measurement of SES in health research: Current practice and steps toward a new approach. Soc. Sci. Med..

[49] Asselin L.M. (2009). Analysis of Multidimensional Poverty: Theory and Case Studies.

[50] Traissac P., Martin-Prevel Y. (2012). Alternatives to principal components analysis to derive asset-based indices to measure socio-economic position in low-and middle-income countries: The case for multiple correspondence analysis. Int. J. Epidemiol..

[51] Sahn D.E., Stifel D.C. (2000). Poverty comparisons over time and across countries in Africa. World Develop..

[52] Howe L.D., Galobardes B., Matijasevich A. (2012). Measuring socio-economic position for epidemiological studies in low-and middle-income countries: A methods of measurement in epidemiology paper. Int. J. Epidemiol..

[53] Statistics South Africa (2011). Census 2011: Census in Brief.

[54] Costa-Font J., Daniele F., Joan G. (2010). Decomposing cross-country differences in levels of obesity and overweight: Does the social environment matter?. Soc. Sci. Med..

[55] Gravelle H. (2003). Measuring income related inequality in health: Standardisation and the partial concentration index. Health Econ..

[56] Wagstaff A., Bilger M., Sajaia Z., Lokshin M. (2011). Health Equity and Financial Protection: Streamlined Anaalysis with ADePT Software.

[57] McKenzie D.J. (2005). Measuring inequality with asset indicators. J. Popul. Econ..

[58] Dutton D.J., McLaren L. (2011). Explained and unexplained regional variation in Canadian obesity prevalence. Obesity.

[59] Finkelstein E.A., Ruhm C.J., Kosa K.M. (2005). Economic causes and consequences of obesity. Annu. Rev. Public Health.

[60] Popkin B.M., Adair L.S., Ng S.W. (2012). Global nutrition transition and the pandemic of obesity in developing countries. Nutr. Rev..

[61] Rudatsikira E., Muula A.S., Mulenga D., Siziya S. (2012). Prevalence and correlates of obesity among Lusaka residents, Zambia: A population-based survey. Int. Arch. Med..

[62] Popkin B.M. (2009). Global changes in diet and a ctivity patterns as drivers of the nutrition transition. Nestle Nutr. Workshop. Ser. Pediatr. Program.

[63] Wardle J., Farrell M., Hillsdon M., Jarvis M.J., Sutton S., Thorogood M., Gordon D., Shaw M., Dorling D., Smith D.G. (1999). Smoking, Drinking, Physical Activity and Screening Uptake And health Inequalities. Inequalities in Health.

[64] (2012). Labour Market Dynamics in South Africa—–2011.

[65] D’Haese M., van Huylenbroeck G. (2005). The rise of supermarkets and changing expenditure patterns of poor rural households case study in the Transkei area, South Africa. Food Policy.

[66] Casale D. (2004). What has the feminisation of the labour market “bought” women in South Africa?: Trends in labour force participation, employment and earnings, 1995–2001. J. Interdisc. Econ..

[67] Micklesfield L.K., Lambert E.V., Hume D.J., Chantler S., Pienaar P.R., Dickie K., Puoane T., Goedecke J.H. (2013). Socio-cultural, environmental and behavioural determinants of obesity in black South African women. Cardiovasc. J. Afr..

[68] Whitaker R.C., Wright J.A., Pepe M.S., Seidel K.D., Dietz W.H. (1997). Predicting obesity in young adulthood from childhood and parental obesity. N. Engl. J. Med..

